# Sex-Stratified Machine Learning for the Prediction of Post-COVID Condition: A Longitudinal Cohort Study

**DOI:** 10.3390/jcm15093367

**Published:** 2026-04-28

**Authors:** Mikhail I. Krivonosov, Ekaterina Pazukhina, Mikhail Rumyantsev, Elina Abdeeva, Dina Baimukhambetova, Polina Bobkova, Yasmin El-Taravi, Maria Pikuza, Anastasia Trefilova, Aleksandr Zolotarev, Margarita Andreeva, Ekaterina Iakovleva, Nikolay Bulanov, Sergey Avdeev, Alexey Zaikin, Valentina Kapustina, Victor Fomin, Andrey A. Svistunov, Peter Timashev, Janna G. Oganezova, Nina Avdeenko, Yulia Ivanova, Lyudmila Fedorova, Elena Kondrikova, Irina Turina, Petr Glybochko, Denis Butnaru, Oleg Blyuss, Daniel Munblit, Sechenov StopCOVID Research Team

**Affiliations:** 1 ISP RAS Research Center for Trusted Artificial Intelligence, 109004 Moscow, Russia; 2 Centre for Cancer Screening, Prevention and Early Diagnosis, Wolfson Institute of Population Health, Queen Mary University of London, London EC1M 6BQ, UK; 3 Department of Pediatrics and Pediatric Infectious Diseases, Institute of Child’s Health, Sechenov First Moscow State Medical University (Sechenov University), 119991 Moscow, Russia; 4 Academician V.I. Krasnopolsky Moscow Regional Research Institute of Obstetrics and Gynecology (MONIIAG), 22a Pokrovka St., 101000 Moscow, Russia; 5 Centre for Health Services and Policy Research, University of British Columbia, Vancouver, BC V6T 1Z4, Canada; 6 Tareev Clinic of Internal Diseases, Sechenov First Moscow State Medical University (Sechenov University), 119435 Moscow, Russia; 7 Clinic of Pulmonology, Sechenov First Moscow State Medical University (Sechenov University), 119991 Moscow, Russia; 8 Institute for Cognitive Neuroscience, University Higher School of Economics, 101000 Moscow, Russia; 9 Department of Mathematics and Women’s Cancer, University College London, London WC1E 6BT, UK; 10 Department of Internal Medicine No. 1, Institute of Clinical Medicine, Sechenov First Moscow State Medical University (Sechenov University), 119435 Moscow, Russia; 11 Sechenov First Moscow State Medical University (Sechenov University), 119991 Moscow, Russia; 12 Institute for Regenerative Medicine, Sechenov First Moscow State Medical University (Sechenov University), 119435 Moscow, Russia; 13 Academician A.P. Nesterov Department of Ophthalmology of the Institute of Clinical Medicine, Pirogov Russian National Research Medical University, 117437 Moscow, Russia; 14 Division of Care in Long Term Conditions, Florence Nightingale Faculty of Nursing, Midwifery and Palliative Care, King’s College London, London WC2R 2LS, UK; 15 Research and Clinical Center for Neuropsychiatry, 119334 Moscow, Russia

**Keywords:** Post-COVID-19 Condition (PCC), sex-specific differences, predictive modeling, longitudinal cohort

## Abstract

**Background**: Post-COVID-19 condition (PCC) affects many survivors, with evidence of sex-specific differences in prevalence and symptom profiles. However, few prediction studies have examined whether sex-stratified models improve prediction or generalize across sexes. This study aimed primarily to develop and compare sex-stratified machine learning models for PCC prediction using routinely available baseline variables, and secondarily to assess cross-sex generalizability and adversarial robustness. **Methods**: We analyzed a prospective longitudinal cohort of 1006 adults hospitalized with COVID-19 at Sechenov University Hospital Network (Moscow, Russia). Demographics, smoking status, and pre-existing comorbidities were extracted from medical records, and PCC status was assessed at 6-month follow-up. Machine learning models—including classical algorithms and graph-based neural networks—were trained separately for males and females. Cross-sex validation evaluated generalizability, variable importance aided interpretation, and adversarial perturbations assessed model robustness. **Results**: PCC prevalence was higher in females (53.9%) than males (39.1%). Overall predictive performance was modest across all models, with AUC values ranging approximately 0.50–0.61. Graph-based models achieved the highest discrimination, with the best AUC reaching approximately 0.61, while classical approaches provided limited predictive value. Cross-sex validation showed minor asymmetry: models trained on male data performed slightly better on female cases than vice versa. Adversarial testing revealed sensitivity of all models to input perturbations. **Conclusions**: Demographics and comorbidities alone provide insufficient information for reliable PCC prediction. Modest sex-specific differences in model generalizability suggest distinct, sex-associated PCC phenotypes, but richer multimodal data—including clinical biomarkers, wearable-derived measures, and patient-reported outcomes—will be required to develop clinically useful and equitable predictive models. Sex-stratified approaches should be considered in future post-viral syndrome prediction studies.

## 1. Introduction

A well-recognized challenge in predicting Post-COVID-19 Condition (PCC), commonly referred to as long COVID, is the significant difference in how it manifests between sexes. The prevalence of PCC is difficult to estimate due to differences in the methodology applied, but some studies report that up to 70% of patients who were previously hospitalized during their initial infection may face PCC in the future [1,2,3]. Importantly, a growing body of observational data consistently demonstrates a female predominance in PCC, with women approximately 1.5 to 2 times more likely than men to experience long-term symptoms [4]. Sex-disaggregated analyses reveal that women and men frequently experience distinct symptom phenotypes in PCC. Females more often report manifestations such as fatigue, neuropsychiatric issues, headaches, joint pain, memory complaints, and sleep disturbances, whereas males may present with differing patterns such as dyspnea or executive dysfunction [5]. A comprehensive review found that females had significantly higher odds of psychiatric, respiratory, musculoskeletal, neurological, dermatological, and gastrointestinal long COVID symptoms compared to males [6]. The importance of sex-disaggregated analysis is further highlighted by the unique risks observed in other viral contexts. For instance, the Zika virus pandemic demonstrated how viral infections can lead to distinct long-term outcomes, such as Congenital Zika Syndrome, which necessitates specific genetic and clinical management strategies [7]. Furthermore, within the female population, symptomatic COVID-19 during pregnancy represents a unique physiological and clinical state that carries significant risk factors and medicolegal implications, emphasizing the need for nuanced data interpretation [8].

Most existing PCC prediction models have pooled men and women or treated sex only as a covariate, and few have tested whether prediction transfers across sexes. This limitation makes it unclear whether sex-specific modeling could improve predictive performance or reveal differences in the relationships between baseline risk factors and later PCC. The primary objective of this study was to develop and compare sex-stratified machine learning models for predicting PCC at 6 months after hospitalization using routinely available baseline demographic, smoking, and comorbidity variables. Secondary objectives were to evaluate cross-sex generalizability and to assess model robustness to small input perturbations. We hypothesized that sex-stratified models might reveal differences in predictive structure and generalizability, although overall discrimination would remain modest because only a limited set of baseline variables was available. In addition, we performed adversarial robustness testing [9,10] as a secondary analysis to evaluate the stability of the developed models under small input perturbations.

## 2. Materials and Methods

### 2.1. Patient Cohort and Data Acquisition

We used data from the longitudinal prospective cohort of adults previously hospitalized with COVID-19 at Sechenov University Hospital Network (Moscow, Russia). Data on demographics and pre-existing comorbidities were extracted from electronic medical records and the local health information system, using the ISARIC WHO Clinical Characterization protocol [11]. A series of follow-up interviews was conducted by a team of trained medical students and physician residents through telephone interviews, with questionnaires filled using the Research Electronic Data Capture (REDCap) database [12,13]. We used the first follow-up interview series, with the average period between hospital discharge and interview of 6 (±2) months.

This study was approved by the Sechenov University Local Ethics Committee on 22 April 2020 (protocol number 08–20, protocol amendment enabling serial follow-up of the cohort was approved on 13 November 2020).

PCC definition was closely aligned with the WHO case definition, defined as the presence of any symptom that began within three months post-hospital discharge and persisted for a minimum of two months [14]. Reported symptoms may involve multiple organ systems and commonly include fatigue, dyspnea, cognitive impairment, muscle or joint pain, sleep disturbance, headache, and anxiety or depression, and are required to have an impact on everyday functioning. Further details on the PCC definition can be found in [15]. Symptom duration was calculated from the hospital discharge date due to the lack of reliable medical records indicating the initial appearance of symptoms. Lack of a recorded symptom during follow-up was interpreted as “no symptom”, therefore no imputations were needed.

We included the following comorbidities, pre-existing at the moment of hospitalization: chronic cardiovascular disease (including coronary insufficiency, heart failure, congenital heart defects, cardiomyopathy and rheumatic heart disease), revascularization of peripheral or coronary arteries, arterial hypertension, chronic pulmonary diseases (including chronic obstructive pulmonary diseases, i.e., chronic bronchitis, chronic obstructive pulmonary disease, emphysema; cystic fibrosis, bronchiectasis, interstitial lung diseases, pre-existing need for long-term oxygen therapy), asthma, chronic kidney disease, obesity (assessed by a clinician, with an objective measurement of obesity, such as calculation of the body mass index of 30 or more or measurement of abdominal girth), moderate to severe liver disease (cirrhosis with portal hypertension, with or without bleeding, or history of variceal bleeding), mild liver diseases (liver cirrhosis without portal hypertension, chronic hepatitis), chronic neurological diseases (cerebral palsy, multiple sclerosis, motor neuron disease, muscular dystrophy, myasthenia gravis, Parkinson’s disease, stroke, severe learning disabilities), malignancies (solid tumors or hematological malignancies excluding malignancies in remission for at least 5 years with no evidence of current disease), rheumatological diseases (inflammatory and degenerative diseases of connective tissue structures including chronic arthropathies and arthritis, connective tissue diseases and vasculitis). We included smoking status as a lifestyle variable, as well as age. These predictors were selected because they were routinely available at baseline and could be extracted consistently across the cohort, allowing a pragmatic low-burden model. The study was not designed as a fully optimized prediction model, and the restricted predictor set likely limited discrimination. No imputation was performed for baseline predictors because the analytic cohort was restricted to participants with complete baseline information on age, smoking status, and comorbidities.

### 2.2. Sex-Stratified Machine Learning Protocol

To assess potential sex-related differences in predictive performance, we applied a sex-stratified modeling strategy. In both symptomatic and asymptomatic settings, the datasets were divided into male and female sub-cohorts. Within each sub-cohort, a 5-fold cross-validation approach was used to evaluate model performance [16]. Variables underwent z-score standardization, with scaling parameters derived exclusively from the training folds within each 5-fold cross-validation split and subsequently applied to both the corresponding training and test folds, to satisfy linear modeling assumptions and harmonize variable scales [17]. No additional feature engineering or data-driven feature selection was performed beyond the prespecified baseline variables and z-score standardization of the continuous predictor.

Five supervised machine learning algorithms were evaluated for their predictive performance: Logistic Regression, Decision Tree, Support Vector Machine (SVM), Random Forest, and the Synolitic Graph Neural Network (SGNN) approach [18]. The SGNN method was included as an exploratory approach to test whether graph-based representations of interactions could improve discrimination from a sparse baseline feature set. The specific configurations, hyperparameter tuning strategies, and cross-validation settings for each algorithm are summarized in Appendix A.1.

Distinct models were trained on male-only and female-only training sets on the same input features. The optimal hyperparameters were selected based on the highest mean AUC across folds. This framework ensured fair and comparable performance assessment across all algorithms.

To assess sex-related algorithmic bias, we designed a rigorous sex-stratified, 5-fold cross-validation (CV) protocol that we applied in each setting separately:

1. The full dataset was first divided into male and female sub-cohorts.
2. Each sub-cohort (male, female) was independently partitioned into 5 matching folds.
3. A series of models was then trained. For each fold k (from 1 to 5): (a) a ‘female-trained’ model was trained on the 4 female folds (k ≠ k), and (b) a ‘male-trained’ model was trained on the 4 male folds (k ≠ k).
4. These trained models were then validated on the held-out k-th folds in all four scenarios: (a) f → f: female-trained model tested on female fold k, (b) f → m: female-trained model tested on male fold k, (c) m → m: male-trained model tested on male fold k, and (d) m → f: male-trained model tested on female fold k.
5. The final performance metrics (AUC, sensitivity, specificity) are the averaged results from across all 5 folds.

Model generalizability was evaluated through four validation scenarios:

- f → f: female-trained model tested on the female cohort (self-validation);
- f → m: female-trained model tested on the male cohort (cross-validation);
- m → m: male-trained model tested on the male cohort (self-validation);
- m → f: male-trained model tested on the female cohort (cross-validation).

Diagnostic performance was quantified primarily using the Area Under the Receiver Operating Characteristic Curve (AUC), supplemented by sensitivity and specificity metrics. Additionally, positive and negative predictive values (PPV, NPV) were calculated for varying prevalence values, and equalized odds differences were computed to assess sex-specific fairness.

For the purpose of this study, the terms ‘male’ and ‘female’ refer to biological sex as recorded in the original dataset. We use ‘sex-stratified’ to describe the analytical framework of training and evaluating separate models for each sex; ‘cross-sex validation’ to describe the evaluation of a model trained on one biological sex and tested on the other (e.g., m → f or f → m), and ‘sex-specific’ to describe patterns, differences, or phenotypes attributable to or associated with one sex. This study did not include data on gender identity or transgender individuals, and ‘cross-sex’ should not be interpreted as a transition-related metric.

All analyses were conducted in R version 4.4.1 using the packages MASS, caret, e1071, randomForest, xgboost, and neuralnet. Age, the only continuous predictor, was standardized prior to model fitting. For the best-performing model, variable importance was assessed using the total reduction in impurity via the varImp function in R. Adversarial robustness analysis was implemented in Python version 3.11.13 using the Adversarial Robustness Toolbox [19,20].

### 2.3. Adversarial Robustness Testing

To assess the stability of our predictive models for potential clinical deployment, we evaluated their robustness against adversarial attacks. This analysis examines how small, controlled input perturbations—mimicking plausible laboratory measurement noise or pre-analytical variability—affect predictive stability. We applied two types of attacks: (1) FGSM (Fast Gradient Sign Method) with ε=0.1 as a mild, single-step attack [21], and (2) PGD (Projected Gradient Descent) with ε=0.3, step size = 0.01, 20 iterations as a strong, iterative attack [22]. We evaluated adversarial robustness because clinical prediction models may be sensitive to small input perturbations, and stability is important for potential deployment.

## 3. Results

In total, 1006 patients were successfully interviewed and included in the analysis. The 6-month follow-up interviews were conducted between 21 November 2020 and 10 October 2021. The overall prevalence of PCC 6 months after hospital discharge reached 46.6%, with 53.9% among females and 39.1% among males. Patients developing PCC were slightly younger than those who did not develop PCC, with a median age of 55 years and 57 years, respectively. The proportion of females was higher among patients with PCC (59%) than among those without PCC (44%). The structure of comorbidities was relatively similar in both. The most common comorbidities in both subsamples were arterial hypertension (47% in the PCC subsample and 45% in non-PCC subsample), chronic cardiovascular diseases and obesity (21% in the PCC subsample and 19% in non-PCC subsample), as well as diabetes (16% in the PCC subsample and 14% in non-PCC subsample). Smoking was slightly more common among patients without PCC (11%) than among those with PCC (8.7%) (Table 1).

To examine the distribution of baseline characteristics by sex, we further analyzed comorbidities, vaccination status, and clinical interventions (Table 2). Female patients exhibited a significantly higher prevalence of several comorbidities compared to males, most notably arterial hypertension (52.9% vs. 37.9%, *p* < 0.001), diabetes (17.6% vs. 11.7%, *p* = 0.01), chronic neurological diseases (6.9% vs. 3.6%, *p* = 0.02), and rheumatological diseases (3.9% vs. 3.0%, *p* = 0.02). However, no statistically significant sex-based differences were observed in influenza or COVID-19 vaccination rates. Similarly, the requirement for acute clinical support, including oxygen therapy, ICU admission, and various modes of ventilation, was comparable between both groups (*p* > 0.05 for all).

Table 3 presents a comparison between the best-performing SGNN model and classical models. Across all evaluated algorithms, predictive performance was generally modest (Table 3 and Table A2), with most models achieving AUC values only slightly above random classification (0.5). Because predictions were generated through fivefold cross-validation with fold-level aggregation, patient-level predicted probabilities from a single held-out sample were not available, precluding the construction of individual ROC curves. Graph-based neural network models (GCN and GATv2) demonstrated the highest discrimination, with AUC values reaching up to 0.61 in some configurations. Classical machine learning models, including logistic regression, decision trees, support vector machines, and random forests, generally showed weaker performance, with AUC values close to random classification.

The SGNN model showed moderate sensitivity to adversarial perturbations (Table 4). Under FGSM (ε = 0.1), the average AUC decreased from 0.589 to 0.503, approaching random classification. Under PGD (ε = 0.3), the reduction was smaller (AUC = 0.536).

Overall, the limited predictive performance observed across models suggests that age, smoking status, and pre-existing comorbidities alone provide insufficient information to reliably predict PCC after hospitalization.

## 4. Discussion

The primary aim of this study was to develop and compare sex-stratified machine learning models for predicting PCC at 6 months after hospitalization using routinely available baseline variables. Despite applying a range of algorithms, predictive performance remained modest, with most models achieving discrimination only slightly above random classification (AUC ≈ 0.55–0.60). These AUC values indicate limited discrimination and fall below what would normally be expected for a clinically deployable prediction tool. Graph-based neural network models achieved the highest AUC values, while classical approaches such as logistic regression, decision trees, support vector machines, and random forests demonstrated similarly limited performance. Our findings suggest that demographic and comorbidity data alone are inadequate for PCC prediction. They are consistent with emerging PCC prediction work. Sudre et al. [23] achieved an AUC of 0.77 for predicting long COVID, but performance depended heavily on first-week symptom counts rather than pre-infection characteristics alone. Jayavelu et al. [24] reported AUROC values of 0.64–0.66 using baseline clinical and immunologic variables, a range comparable to our own models. Fjelltveit et al. [25], using comprehensive pre-infection register data including healthcare utilization and vaccination status, achieved an AUC of 0.78, suggesting that substantially richer feature sets are required to move prediction beyond modest discrimination. Our findings extend this literature by testing sex-stratified modeling and cross-sex generalizability within a hospitalized cohort. While some differences were observed between sexes, these could reflect model instability or limited feature sets rather than true biological divergence. The results suggest that increasing model complexity did not overcome the limited information content of the predictor set, and simpler interpretable approaches may perform comparably in this setting. Future work should aim to integrate clinical biomarkers (e.g., inflammatory markers like CRP, D-dimer), data from wearables (e.g., activity levels, heart rate variability), and patient-reported outcome measures to build a more holistic and predictive model of PCC.

The observed sex-related differences are hypothesis-generating rather than definitive evidence of distinct biological phenotypes. They may reflect true sex-related differences, but they may also be explained by sampling variability, limited predictors, or model instability. When trained on female data, both decision tree and random forest models performed worse in predicting outcomes among males, whereas male-trained models performed slightly better when applied to female datasets. This asymmetry may reflect sex-specific pathophysiological or behavioral differences in PCC development, although it could also arise from model instability or the limited set of predictors available in this study. A critical dimension of this asymmetry may involve the psychological implications of PCC. Females in our study reported a higher prevalence of neuropsychiatric symptoms, which aligns with broader evidence of the significant psychological burden associated with post-viral recovery. Addressing these implications including how mental health intersects with physical symptoms and patient-reported outcomes is essential for understanding the holistic risk profile of patients and improving the predictive power of future AI models. These findings highlight the importance of considering demographic variables such as sex when developing clinical AI models, as pooling heterogeneous populations may obscure meaningful differences in model behavior across subgroups. Our findings may have implications beyond PCC, suggesting that sex-stratified modeling should be a primary consideration in the development of predictive tools for other complex, heterogeneous conditions where sex-specific pathophysiology is suspected, such as in autoimmune disorders or ME/CFS.

Our findings have several implications. First, they suggest that demographic and comorbidity variables, while important for characterizing patient populations, are insufficient standalone predictors for PCC risk. More granular data—including clinical biomarkers, immunological profiles, radiological findings, wearable-derived measures, and psychosocial indicators—are likely required. Second, the observed sex asymmetry in model generalizability highlights the need for sex-sensitive approaches in PCC research, with careful attention to potential biological and sociocultural differences. Furthermore, the clinical landscape of post-viral conditions is influenced by external behavioral and systemic factors. For example, factors such as vaccine acceptance among high-risk groups, including pregnant women, play a crucial role in shaping long-term immunity and outcomes [26]. Additionally, shifts in obstetric and gynecological hospitalizations during pandemic-related restrictive measures suggest that healthcare-seeking behaviors and system pressures may indirectly impact the recovery and diagnosis of PCC in women [27]. Third, the limited robustness of models underscores the importance of incorporating adversarial validation into clinical machine learning studies, as apparent predictive success on observational data may not translate into reliable performance in more diverse or perturbed contexts.

This study has limitations. The sample consisted of patients hospitalized during the first wave of COVID-19 in Moscow, which may not generalize to later variants or to patients with milder acute disease. PCC was assessed through telephone follow-up, which, while practical, may be subject to recall bias and misclassification. The restricted set of predictors also means that important acute-phase determinants of PCC may have been omitted, which likely limited predictive performance. Absence of a recorded symptom at follow-up was treated as absence of that symptom. Although this approach may under-ascertain symptoms occurring outside the follow-up window, interviews were comprehensive and consistently administered, making this assumption reasonable for the purposes of the present analysis.

We intentionally adopted a sex-stratified modeling framework rather than including sex as a covariate in a pooled model, as this approach allows for differences in model structure and predictor importance between sexes to emerge more clearly.

In conclusion, demographics and comorbidity variables alone do not provide a clinically useful prediction of PCC. The observed sex-related differences are insufficient to confirm distinct sex-specific phenotypes and should be treated as hypothesis-generating.

## Figures and Tables

**Table 1:** Descriptive characteristics of PCC and non-PCC patients.

Characteristic	No PCC	PCC	*p*-Value ^2^
	N = 537 ^1^	N = 469 ^1^	
Age	57 (47, 67)	55 (47, 64)	0.2
Sex at birth			<0.001
female	235 (44%)	275 (59%)	
male	302 (56%)	194 (41%)	
Chronic cardiovascular diseases	101 (19%)	97 (21%)	0.5
Arterial hypertension	239 (44.5%)	219 (46.7%)	0.527
Revascularization of peripheral and/or coronary arteries	32 (6.0%)	19 (4.1%)	0.2
Chronic pulmonary diseases	39 (7.3%)	40 (8.5%)	0.5
Asthma	23 (4.3%)	25 (5.3%)	0.4
Chronic kidney disease	26 (4.8%)	25 (5.3%)	0.7
Obesity	101 (19%)	97 (21%)	0.5
Moderate to severe liver disease	8 (1.5%)	2 (0.4%)	0.12
Mild liver diseases	11 (2.0%)	6 (1.3%)	0.3
Chronic neurological diseases	32 (6.0%)	21 (4.5%)	0.3
Malignancies	25 (4.7%)	17 (3.6%)	0.4
Diabetes	74 (14%)	74 (16%)	0.4
Rheumatological diseases	13 (2.4%)	14 (3.0%)	0.6
Smoking	58 (10.8%)	41 (8.7%)	0.3

^1^ n (%); Median (Q1, Q3). ^2^ Pearson’s Chi-squared test for categorical variables with any expected cell count above or equal to 5; Wilcoxon rank sum test for continuous variables with two levels; Fisher’s exact test for categorical variables with any expected cell count below 5.

**Table 2:** Sex-stratified baseline characteristics of the study population.

Characteristic	Female	Male	*p*-Value ^2^
	N = 510 ^1^	N = 496 ^1^	
Chronic cardiovascular diseases	106 (20.8%)	92 (18.5%)	0.4
Arterial hypertension	270 (52.9%)	188 (37.9%)	<0.001
Revascularization of peripheral and/or coronary arteries	23 (4.5%)	28 (5.6%)	0.5
Chronic pulmonary diseases	36 (7.1%)	43 (8.7%)	0.4
Asthma	30 (5.9%)	18 (3.6%)	0.1
Chronic kidney disease	32 (6.3%)	19 (3.8%)	0.1
Obesity	109 (21.4%)	89 (17.9%)	0.2
Moderate to severe liver disease	7 (1.4%)	3 (0.6%)	0.3
Mild liver diseases	8 (1.6%)	9 (1.8%)	0.8
Chronic neurological diseases	35 (6.9%)	18 (3.6%)	0.02
Malignancies	25 (4.9%)	17 (3.4%)	0.3
Diabetes	90 (17.6%)	58 (11.7%)	0.01
Rheumatological diseases	20 (3.9%)	7 (1.4%)	0.02
Influenza vaccination	96 (19.8%)	101 (20.9%)	0.7
COVID-19 vaccination	64 (12.9%)	68 (14.1%)	0.6
Oxygen therapy	177 (35.5%)	188 (38.5%)	0.4
Non-invasive ventilation	6 (1.2%)	6 (1.2%)	1
Invasive ventilation	2 (0.4%)	0 (0%)	0.5
ICU	13 (2.6%)	7 (1.4%)	0.3
Prone ventilation	71 (14.2%)	87 (17.9%)	0.1
Tracheostomy	3 (0.6%)	0 (0%)	0.2
Vasopressor treatment	1 (0.2%)	0 (0%)	1

^1^ n (%). ^2^ Pearson’s Chi-squared test for categorical variables with any expected cell count above or equal to 5; Fisher’s exact test for categorical variables with any expected cell count below 5.

**Table 3:** Results of sex-stratified AI models.

Model Type	Sparsity	Specification	Sensitivity	Specificity	AUC
GATv2	None	f → f	0.542	0.638	0.582
Node feature = False		f → m	0.557	0.656	0.587
		m → m	0.727	0.490	0.592
		m → f	0.633	0.579	0.596
Logistic regression		f → f	0.601	0.537	0.536
		f → m	0.728	0.463	0.566
		m → m	0.63	0.577	0.569
		m → f	0.584	0.591	0.562
Decision tree		f → f	0.406	0.659	0.532
		f → m	0.395	0.691	0.543
		m → m	0.816	0.214	0.515
		m → f	0.811	0.217	0.514
SVM		f → f	0.109	0.912	0.51
		f → m	0.078	0.942	0.51
		m → m	0.946	0.028	0.487
		m → f	0.933	0.07	0.501
Random forest		f → f	0.331	0.687	0.509
		f → m	0.276	0.777	0.526
		m → m	0.883	0.108	0.496
		m → f	0.902	0.162	0.532
Lasso		f → f	1	0	0.498
		f → m	0.995	0.007	0.501
		m → m	1	0	0.496
		m → f	0.81	0.191	0.501

**Table 4:** GATv2 SGNN adversarial robustness results.

Model Type	Sparsity	Specification	Clean AUC	PGD AUC	FGSM AUC
GATv2	None	f → f	0.582 ± 0.062	0.536 ± 0.061	0.466 ± 0.122
Node feature = False		f → m	0.587 ± 0.064	0.524 ± 0.042	0.504 ± 0.035
		m → m	0.592 ± 0.048	0.550 ± 0.058	0.525 ± 0.036
		m → f	0.596 ± 0.076	0.535 ± 0.072	0.515 ± 0.071
Average			0.589	0.536	0.503

## Data Availability

Data available upon reasonable request.

## Appendix A

### Appendix A.1

The implementation details, including architectural parameters and tuning protocols for the suite of machine learning approaches used in this analysis, are delineated below.

- Logistic Regression (LR): Implemented in both standard and L1-regularized (LASSO) forms to assess linear model performance with and without regularization.
- Decision Tree (DT): Models were fit using a cost-complexity pruning strategy. Trees were initially grown with relaxed constraints (complexity parameter cp = 0) to capture potential non-linear interactions, followed by post-pruning to the optimal cp value that minimized cross-validation error, thereby ensuring model stability and preventing overfitting.
- Support Vector Machine (SVM): Models were trained using a radial basis function (RBF) kernel ($kernel\;=\prime radial^{\prime }$) to effectively model non-linear decision boundaries. Hyperparameter tuning was performed via grid search over both the regularization parameter (C) and the kernel coefficient (γ) using 5-fold cross-validation within the training set.
- Random Forest (RF): Models were fit with 500 trees (ntree = 500). To optimize performance, the number of variables considered at each split (mtry) was tuned using the tuneRF function, which selects the optimal value based on the minimization of Out-of-Bag (OOB) error estimates.
- Synolitic Graph Neural Network (SGNN): For each patient sample, a weighted graph is constructed where nodes represent variables. Edge weights between nodes i and j are determined by the probability output of a Support Vector Machine (SVM) classifier trained on those two features:

$$w_{ij}=P(\mathrm{PCC}|x_{i},x_{j})$$

Four graph sparsification strategies have been evaluated:

○ No sparsification: All edges retained;
○ Sparsify *p* = 0.2: Keep top 20% of edges ranked by $\left|w_{ij}-0.5\right|$;
○ Sparsify *p* = 0.8: Keep top 80% of edges ranked by $\left|w_{ij}-0.5\right|$;
○ Minimum connected: Remove edges while maintaining graph connectivity.

Together with two node feature configurations:

○ Basic features: Standardized variable values only;
○ Graph-based features: Standardized values plus node degree, weighted degree (strength), closeness centrality, and betweenness centrality.

The Graph Convolutional Network (GCN) architecture consists of:

○ Two GCN layers with 32 hidden channels each;
○ LeakyReLU activation, batch normalization, and dropout (0.3);
○ Global mean pooling for graph-level representation;
○ Linear classifier for binary classification.

The model was trained using the AdamW optimizer with a learning rate of 0.001 and weight decay of $10^{-4}$, with early stopping (patience = 15) based on validation AUC.

**Table A1:** Summary of machine learning model configurations.

Model Type	Key Hyperparameters	Tuning Strategy	Cross-Validation Settings
Logistic Regression (Standard & L1)	L1 penalty for regularized variant	Standard coefficients; L1 assessed for stability	5-fold stratified CV
Decision Tree (DT)	Complexity parameter (cp)	Cost-complexity post-pruning based on minimum CV error	5-fold stratified CV
Support Vector Machine (SVM)	Radial Basis Function (RBF) kernel; C and γ	Grid search over C and γ	5-fold inner CV for tuning; 5-fold outer CV for testing
Random Forest (RF)	ntree = 500; mtry	mtry optimized via tuneRF to minimize OOB error	5-fold stratified CV

### Appendix A.2

The performance of all SGNN configurations is shown below.

**Table A2:** Results of sex specific SGNN models.

Model Type	Sparsity	Specification	Sensitivity	Specificity	AUC
GCN	None	f → f	0.785	0.421	0.562
Node feature = True		f → m	0.531	0.669	0.580
		m → m	0.634	0.569	0.578
		m → f	0.516	0.677	0.578
	*p* = 0.2	f → f	0.680	0.557	0.576
		f → m	0.610	0.587	0.589
		m → m	0.491	0.729	0.567
		m → f	0.607	0.634	0.553
	*p* = 0.5	f → f	0.702	0.430	0.526
		f → m	0.572	0.659	0.590
		m → m	0.557	0.620	0.567
		m → f	0.495	0.711	0.587
	*p* = 0.8	f → f	0.604	0.591	0.569
		f → m	0.602	0.598	0.572
		m → m	0.444	0.766	0.576
		m → f	0.520	0.630	0.551
GCN	None	f → f	0.647	0.553	0.583
Node feature = False		f → m	0.708	0.468	0.542
		m → m	0.640	0.554	0.568
		m → f	0.604	0.609	0.591
	*p* = 0.2	f → f	0.691	0.502	0.570
		f → m	0.552	0.672	0.586
		m → m	0.726	0.443	0.558
		m → f	0.604	0.587	0.586
	*p* = 0.5	f → f	0.756	0.400	0.543
		f → m	0.577	0.636	0.575
		m → m	0.594	0.594	0.559
		m → f	0.618	0.562	0.562
	*p* = 0.8	f → f	0.589	0.643	0.605
		f → m	0.696	0.495	0.567
		m → m	0.625	0.548	0.538
		m → f	0.400	0.783	0.568
GATv2	None	f → f	0.538	0.698	0.584
Node feature = True		f → m	0.718	0.530	0.582
		m → m	0.737	0.443	0.574
		m → f	0.680	0.536	0.601
	*p* = 0.2	f → f	0.644	0.557	0.574
		f → m	0.685	0.496	0.571
		m → m	0.661	0.570	0.595
		m → f	0.669	0.536	0.589
	*p* = 0.5	f → f	0.491	0.740	0.615
		f → m	0.551	0.636	0.579
		m → m	0.409	0.775	0.553
		m → f	0.455	0.728	0.570
	*p* = 0.8	f → f	0.436	0.757	0.581
		f → m	0.511	0.670	0.575
		m → m	0.490	0.703	0.578
		f → f	0.698	0.489	0.570
GATv2	None	f → f	0.542	0.638	0.582
Node feature = False		f → m	0.557	0.656	0.587
		m → m	0.727	0.490	0.592
		m → f	0.633	0.579	0.596
	*p* = 0.2	f → f	0.527	0.643	0.558
		f → m	0.742	0.426	0.575
		m → m	0.701	0.530	0.604
		m → f	0.578	0.647	0.595
	*p* = 0.5	f → f	0.596	0.609	0.588
		f → m	0.531	0.650	0.571
		m → m	0.480	0.716	0.574
		m → f	0.524	0.664	0.577
	*p* = 0.8	f → f	0.727	0.362	0.493
		f → m	0.423	0.748	0.563
		m → m	0.531	0.656	0.568
		f → f	0.582	0.651	0.607

## References

[1] Carfì A., Bernabei R., Landi F. (2020). Persistent Symptoms in Patients After Acute COVID-19. JAMA.

[2] Fankuchen O., Lau J., Rajan M.M., Swed B., Martin P., Hidalgo M., Yamshon S., Pinheiro L., Shah M.A. (2023). Long COVID in Cancer: A Matched Cohort Study of 1-year Mortality and Long COVID Prevalence Among Patients with Cancer Who Survived an Initial Severe SARS-CoV-2 Infection. Am. J. Clin. Oncol..

[3] Martimbianco A.L.C., Pacheco R.L., Bagattini Â.M., Riera R. (2021). Frequency, signs and symptoms, and criteria adopted for long COVID-19: A systematic review. Int. J. Clin. Pract..

[4] Tsampasian V., Elghazaly H., Chattopadhyay R., Debski M., Naing T.K.P., Garg P., Clark A., Ntatsaki E., Vassiliou V.S. (2023). Risk Factors Associated with Post-COVID-19 Condition: A Systematic Review and Meta-analysis. JAMA Intern. Med..

[5] Delfino C., Poli M.C., Vial C., Vial P.A., Martínez G., Riviotta A., Arbat C., Mac-Guire N., Hoppe J., Carvajal C. (2024). Post-COVID-19 condition: A sex-based analysis of clinical and laboratory trends. Front. Med..

[6] Sylvester S.V., Rusu R., Chan B., Bellows M., O’keefe C., Nicholson S. (2022). Sex differences in sequelae from COVID-19 infection and in long COVID syndrome: A review. Curr. Med. Res. Opin..

[7] Gullo G., Scaglione M., Cucinella G., Riva A., Coldebella D., Cavaliere A.F., Signore F., Buzzaccarini G., Spagnol G., Laganà A.S. (2022). Congenital Zika Syndrome: Genetic Avenues for Diagnosis and Therapy, Possible Management and Long-Term Outcomes. J. Clin. Med..

[8] Maranto M., Zaami S., Restivo V., Termini D., Gangemi A., Tumminello M., Culmone S., Billone V., Cucinella G., Gullo G. (2023). Symptomatic COVID-19 in Pregnancy: Hospital Cohort Data between May 2020 and April 2021, Risk Factors and Medicolegal Implications. J. Clin. Med..

[9] Szegedy C., Zaremba W., Sutskever I., Bruna J., Erhan D., Goodfellow I., Fergus R. Intriguing Properties of Neural Networks. Proceedings of the International Conference on Learning Representations (ICLR).

[10] Brendel W., Carlini N., Tramer F., Zimmermann R.S. (2022). Increasing confidence in adversarial robustness evaluations. Adv. Neural Inf. Process. Syst..

[11] The World Health Organization (2023). Statement on the Update of WHO’s Working Definitions and Tracking System for SARS-CoV-2 Variants of Concern and Variants of Interest. https://www.who.int/news/item/16-03-2023-statement-on-the-update-of-who-s-working-definitions-and-tracking-system-for-sars-cov-2-variants-of-concern-and-variants-of-interest.

[12] Munblit D., Bobkova P., Spiridonova E., Shikhaleva A., Gamirova A., Blyuss O., Nekliudov N., Bugaeva P., Andreeva M., DunnGalvin A. (2021). Incidence and risk factors for persistent symptoms in adults previously hospitalized for COVID-19. Clin. Exp. Allergy.

[13] Munblit D., Nekliudov N.A., Bugaeva P., Blyuss O., Kislova M., Listovskaya E., Gamirova A., Shikhaleva A., Belyaev V., Timashev P. (2021). Stop COVID Cohort: An Observational Study of 3480 Patients Admitted to the Sechenov University Hospital Network in Moscow City for Suspected Coronavirus Disease 2019 (COVID-19) Infection. Clin. Infect. Dis..

[14] Soriano J.B., Murthy S., Marshall J.C., Relan P., Diaz J.V. (2022). A clinical case definition of post-COVID-19 condition by a Delphi consensus. Lancet Infect. Dis..

[15] Pazukhina E., Andreeva M., Spiridonova E., Bobkova P., Shikhaleva A., El-Taravi Y., Rumyantsev M., Gamirova A., Bairashevskaia A., Petrova P. (2022). Sechenov StopCOVID Research Team Prevalence and risk factors of post-COVID-19 condition in adults and children at 6 and 12 months after hospital discharge: A prospective, cohort study in Moscow (StopCOVID). BMC Med..

[16] Steyerberg E.W., Vergouwe Y. (2014). Towards better clinical prediction models: Seven steps for development and an ABCD for validation. Eur. Heart J..

[17] Looney S.W., Hagan J.L., Chen D.-G. (2024). Statistical Challenges in the Analysis of Biomarker Data. Biostatistics in Biopharmaceutical Research and Development: Clinical Trial Analysis.

[18] Zaikin A., Sviridov I., Oganezova J.G., Menon U., Gentry-Maharaj A., Timms J.F., Blyuss O. (2025). Synolitic Graph Neural Networks of High-Dimensional Proteomic Data Enhance Early Detection of Ovarian Cancer. Cancers.

[19] Lundberg S.M., Lee S.-I. A unified approach to interpreting model predictions. Proceedings of the 31st Conference on Neural Information Processing Systems (NIPS 2017).

[20] Nicolae M.-I., Sinn M., Tran M.N., Buesser B., Rawat A., Wistuba M., Zantedeschi V., Baracaldo N., Chen B., Ludwig H. (2018). Adversarial Robustness Toolbox v1. 0.0. arXiv.

[21] Goodfellow I.J., Shlens J., Szegedy C. (2015). Explaining and Harnessing Adversarial Examples. International Conference on Learning Representations (ICLR). arXiv.

[22] Madry A., Makelov A., Schmidt L., Tsipras D., Vladu A. (2018). Towards Deep Learning Models Resistant to Adversarial Attacks. arXiv.

[23] Sudre C.H., Murray B., Varsavsky T., Graham M.S., Penfold R.S., Bowyer R.C., Pujol J.C., Klaser K., Antonelli M., Canas L.S. (2021). Attributes and predictors of long COVID. Nat. Med..

[24] Jayavelu N.D., Samaha H., Wimalasena S.T., Hoch A., Gygi J.P., Gabernet G., Ozonoff A., Liu S., Milliren C.E., Levy O. (2026). Machine learning models predict long COVID outcomes based on baseline clinical and immunologic factors. Commun. Med..

[25] Reme B.-A., Gjesvik J., Magnusson K. (2023). Predictors of the post-COVID condition following mild SARS-CoV-2 infection. Nat. Commun..

[26] Maranto M., Gullo G., Bruno A., Minutolo G., Cucinella G., Maiorana A., Casuccio A., Restivo V. (2023). Factors Associated with Anti-SARS-CoV-2 Vaccine Acceptance among Pregnant Women: Data from Outpatient Women Experiencing High-Risk Pregnancy. J. Clin. Med..

[27] Riemma G., De Franciscis P., Tesorone M., Coppa E., Schiattarella A., Billone V., Lopez A., Cucinella G., Gullo G., Carotenuto R.M. (2023). Obstetric and Gynecological Admissions and Hospitalizations in an Italian Tertiary-Care Hospital during COVID-19 Pandemic: A Retrospective Analysis According to Restrictive Measures. J. Clin. Med..

